# Bridging the gap: ctDNA, genomics, and equity in breast cancer care

**DOI:** 10.1038/s41523-025-00811-1

**Published:** 2025-08-16

**Authors:** Julia Aronson, Manasa Bhatta, Lisa A. Carey, Kunal Jobanputra, Gaorav P. Gupta, Yara Abdou

**Affiliations:** 1 Department of Medicine, Division of Oncology, UNC Chapel Hill, Chapel Hill, NC USA; 2 Department of Medical Oncology, MOC Cancer Care & Research Centre, Mumbai, India; 3 Department of Radiation Oncology, UNC Chapel Hill, Chapel Hill, NC USA

**Keywords:** Population screening, Breast cancer

## Abstract

Circulating tumor DNA (ctDNA) has emerged as a powerful tool in precision oncology, offering a noninvasive approach to tumor profiling, minimal residual disease (MRD), and treatment monitoring. In breast cancer, ctDNA has shown promise in both metastatic and early-stage settings. However, its application and benefits have not been equitably realized across all populations. In this review, we examined the current evidence on ctDNA detection, assay performance, and clinical utility specifically within racially, ethnically, and geographically underrepresented populations. We synthesized data from genomic studies, ctDNA-based trials, and implementation research to identify disparities in ctDNA levels, mutational profiles, testing utilization, and access to genotype-matched therapies. These disparities were further compounded by structural barriers such as insurance coverage, geographic access, and limited inclusion in clinical research. Global data from low- and middle-income countries reinforced both the feasibility and the challenges of ctDNA implementation in resource-constrained settings. While ctDNA holds considerable potential to personalize breast cancer care, our findings underscore the urgent need to integrate equity into its validation, clinical application, and policy development to avoid perpetuating existing disparities.

## Introduction

Circulating tumor DNA (ctDNA), consisting of small fragments of tumor-derived DNA released into the bloodstream, has emerged as a promising biomarker in oncology. Its known and potential applications span early detection, prognostication, minimal residual disease (MRD) assessment, non-invasive tumor genotyping, and monitoring of treatment response. Because ctDNA analysis is noninvasive and can be performed serially, it holds promise for broad use in clinical practice, offering a path toward more personalized and dynamic cancer care^1,2^.

In breast cancer, ctDNA has demonstrated utility across multiple clinical contexts. It has become a mainstay in the management of metastatic breast cancer (MBC) with its ability to detect genetic alterations, such as ESR1, PIK3CA, and PTEN mutations, that may inform prognosis while also serving as a predictive biomarker to guide therapeutic decision-making^3–7^. Also compelling is the ability of ctDNA to detect MRD after curative intent therapy, enabling the early identification of patients at high risk of recurrence and potentially informing treatment escalation or de-escalation—although its clinical utility in guiding patient management decisions has yet to be established^7–12^.

However, while ctDNA represents a major advance in precision oncology, its equitable implementation remains a challenge. The integration of ctDNA technologies faces inherent challenges in use across a variety of practice settings and populations due to availability in pathology labs and variable insurance coverage of biomarker testing^1,13^. This is particularly concerning in breast cancer, where striking racial and ethnic disparities in outcomes persist^14–16^.

Black women, for instance, are disproportionately affected by aggressive subtypes such as basal-like or triple-negative breast cancer (TNBC), with non-Hispanic Black women being nearly twice as likely to be diagnosed with TNBC compared to non-Hispanic white women^14,17,18^. Black women also have a higher risk of breast cancer death compared to White women in all breast tumor subtypes and poorer overall survival for all subtypes^14,19^, a gap driven by complex, interrelated factors including delayed diagnosis, unequal access to care, structural racism, and socioeconomic challenges^20–24^. These outcome gaps are further exacerbated by the persistent underrepresentation of Black patients in cancer clinical trials^25,26^, with Black patients comprising only 7.44% of participants in trials supporting FDA approvals of cancer therapeutics between 2014 and 2018^27^. This lack of representation raises critical concerns about the generalizability of emerging precision oncology tools, such as ctDNA-based biomarkers and algorithms, which may not perform equally across all populations.

Despite the promise of ctDNA and genomic profiling to tailor cancer care, the absence of inclusive research threatens to widen existing inequities. Structural barriers, including cost, inconsistent insurance coverage, and limited geographic access to advanced molecular diagnostics, disproportionately affect racial and ethnic minoritized populations, limiting their access to these emerging tools^1^.

In this review, we focus on the current body of evidence surrounding ctDNA detection, performance, and clinical application, specifically in racially, ethnically, and geographically underrepresented populations with breast cancer. We explore how biological variability and structural inequities may contribute to differences in assay sensitivity, utilization, and downstream treatment opportunities. By synthesizing these findings, we aim to underscore the urgent need for equity-focused research, validation, and implementation strategies to ensure that ctDNA-based tools benefit all patients, not just those historically overrepresented in precision oncology studies.

## ctDNA detection and assay performance across diverse populations

Current ctDNA detection technologies primarily include next-generation sequencing (NGS), digital PCR, and tumor-informed approaches. NGS enables broad genomic profiling across multiple regions, while digital PCR offers high sensitivity and specificity for detecting predefined mutations. Tumor-informed assays, which personalize ctDNA analysis using mutations identified in a patient’s own tumor tissue, offer improved specificity and clinical relevance^2,28^, making them particularly well-suited for MRD detection. However, these assays may not capture newly acquired mutations, limiting their utility for tracking tumor evolution over time.

Biological variability in ctDNA shedding and clearance may influence the performance of these assays across different patient populations. Tumors with high proliferative activity, such as TNBC, tend to release more ctDNA due to increased cellular turnover, thereby increasing the likelihood of detection^29^. In contrast, tumors with low burden or indolent behavior may shed minimal ctDNA, limiting assay sensitivity^30^. The tumor microenvironment, including factors like stromal density and vascular supply, may also influence ctDNA release, further complicating detection efforts^31^.

Clearance of ctDNA from circulation is another potential source of variability. ctDNA half-life, generally estimated at 1–2 hours, is affected by hepatic and renal function, immune clearance, and underlying metabolic conditions^28,32^. These parameters may differ across racial and ethnic groups due to differences in comorbidities. For example, metabolic syndrome, more prevalent in some minority populations, could affect ctDNA kinetics and lead to differences in assay sensitivity and interpretation^33,34^. One analysis demonstrated that patients of African ancestry have significantly higher ctDNA positivity rates and ctDNA levels compared to patients of other ancestries, even after adjusting for disease stage, suggesting that ancestry-related biological differences may influence ctDNA shedding and interpretation^35^.

Furthermore, disparities in the utilization of ctDNA testing have been observed in clinical practice. In one study of patients with recurrent MBC, individuals who were not Hispanic or Latino had four times higher odds of receiving NGS (whether tissue- or ctDNA-based testing) compared to those who were Hispanic or Latino^36^. These findings are consistent with another real-world study showing lower-than-expected rates of ctDNA testing among Hispanic patients with breast cancer, with an observed to expected ratio of ctDNA testing to incidence of 0.80 (confidence interval (CI) 0.77–0.83)^37^. Reassuringly, some studies in breast cancer suggest comparable rates of biomarker testing between non-Hispanic White and Black patients^36^; however, lower testing rates among Black patients have been observed in other malignancies^37,38^.

Disparities in detection sensitivity and utilization may contribute to unequal clinical outcomes. Reduced assay performance in underrepresented populations can result in delayed detection, underestimation of MRD, and suboptimal treatment monitoring. Without data that reflect racially and ethnically diverse populations, these limitations risk reinforcing existing inequities in cancer care and outcomes. Conversely, if ctDNA-based biomarkers outperform other commonly used biomarkers in select breast cancer populations, broader adoption may offer a strategy to reduce disparities. A deeper understanding of how biological and systemic factors affect ctDNA dynamics across populations is essential to ensure equitable application of this technology.

## Racial and ethnic variation in ctDNA genomic profiles and clinical application

Emerging evidence suggests that both the genomic profiles captured by ctDNA and the way ctDNA findings are used in clinical care may vary across racial and ethnic groups^39,40^. While ctDNA generally reflects the mutational landscape observed in tumor tissue, few studies have systematically compared ctDNA characteristics across racial populations. Even fewer have explored how these biological differences may interact with disparities in clinical utilization, including whether patients receive matched targeted therapies or are enrolled in ctDNA-informed clinical trials. This dual gap in our understanding of both ctDNA biology and its downstream clinical application raises important questions about whether ctDNA is being used equitably to guide care in diverse patient populations^41,42^.

A recent analysis from Podany et al. explored ctDNA profiles in Black and White patients with MBC and identified notable differences. Black patients had higher frequencies of single-nucleotide variants (SNVs) in CDKN2 (OR 5.37), GATA3 (OR 1.99), and PTPN11 (OR 7.96), and copy number variations (CNVs) in CCND2 (OR 3.36) compared to White patients. In terms of pathway-level alterations, Black patients most commonly had alterations in TP53 SNV (47.4%), PI3K SNV (31.8%), receptor tyrosine kinase CNV (27.4%), ER SNV (26.7%), MYC CNV (11.1%), and RAF CNV (8.9%). In a separate database, Black patients had significantly higher ctDNA frequencies of TP53, GATA3, CCND2, FGFR1, CCNE1, MYC, ERBB2, and KRAS alterations, whereas White patients more frequently harbored alterations in PIK3CA, ATM, EGFR, BRCA2, CDH1, MET, KRAS, and CHEK2^39^.

In another study of patients with stage I to III breast cancer, Black patients had significantly higher rates of TP53 mutations and significantly lower rates of PIK3CA mutations compared to White patients, though no differences were seen in GATA3 mutations. Greater intratumor heterogeneity and more frequent TP53 mutations have been identified as contributors to recurrence and poor prognosis in Black patients^43^. These somatic differences have been replicated in other genomic analyses, further supporting the pattern of elevated TP53 mutation rates and reduced PIK3CA alteration frequencies in breast tumors from Black patients^44–46^. Notably, emerging data also suggest that patients with TP53-mutated tumors may have higher ctDNA levels and are more likely to be ctDNA positive, even at similar disease stages, compared to patients with non-TP53 mutated tumors^35^.

Extending beyond breast cancer, higher TP53 mutation frequencies and lower PI3K pathway alterations among Black patients have also been observed in pan-cancer analyses, suggesting that such disparities may reflect broader biological patterns across malignancies^47,48^. These somatic differences may, in part, be driven by underlying germline predisposition. In one study investigating the relationship between germline and somatic profiles, Black women were significantly more likely to carry germline BRCA1 mutations compared to other groups (*p* = 0.008). Within this cohort, tumors from BRCA1, BRCA2, and PALB2 carriers exhibited higher rates of somatic TP53 mutations, while those from ATM and CHEK2 carriers had lower frequencies of TP53 alterations^49^. These findings may help explain the enrichment of TP53 mutations among Black patients and underscores the importance of considering both inherited and tumor-specific genomic differences when evaluating disparities in breast cancer biology.

An important and emerging concern is whether racial disparities exist not only in genomic profiling, but also in the overall clinical utilization of precision oncology, including the application of genomic findings to guide therapy selection. Podany et al. investigated racial differences in the use of targeted therapies, focusing particularly on the use of PI3K, mTOR, and CDK4/6 inhibitors^39^. Among patients with PI3KCA mutations, Black patients were significantly less likely to receive PI3K inhibitors, with higher baseline HbA1c levels suggested as a possible contributing factor. No differences were noted in the use of CDK4/6 or mTOR inhibitors, both of which do not require mutation-based selection. These findings suggest that disparities may be more pronounced for genomically matched therapies. Alarmingly, the study also found that none of the Black patients with PIK3CA SNVs were enrolled in a corresponding clinical trial, compared to 11.5% of White patients. Moreover, the presence of PI3K pathway alterations was associated with worse prognosis in Black patients with HR+, HER2-negative breast cancer, underscoring the clinical impact of underutilizing genotype-directed therapy in this population. Among all patients tested, overall survival from the time of ctDNA collection was significantly worse for Black patients in multivariable-adjusted analyses^39^. Supporting these findings, a separate cohort study of 425 patients with MBC showed that White patients who underwent cell-free DNA testing were more likely to receive matched targeted therapies compared to Black, Hispanic, and Asian patients, despite similar rates of actionable mutations^40^. Together, these findings suggest that racial disparities in the application of ctDNA results may be an underrecognized but significant driver of outcome differences.

## Global Insights from Underrepresented Populations

Emerging data from low- and middle-income countries (LMIC) also highlight the potential role of ctDNA in addressing global disparities in breast cancer care. In Ghana, where breast cancer incidence is increasing and TNBC is more common than in European populations, late-stage presentation is frequent, and 62% of patients are diagnosed with tumors larger than 5 cm, reflecting both delayed detection and aggressive disease biology^50^. In a small study, Ahuno et al. evaluated the feasibility of ctDNA analysis using whole-genome sequencing (WGS) of cell-free DNA from 15 breast cancer patients at Korle-Bu Teaching Hospital in Accra, Ghana^51^. ctDNA was detectable in all patients using high-depth (30x) WGS, and in 80% of patients using low-depth (1x) WGS, demonstrating that ctDNA profiling may be feasible even in resource-limited settings. TNBC cases had significantly higher ctDNA levels compared to non-TNBC cases (*p* = 0.04), and recurrent copy number gains were identified at key oncogenic loci, including ZNF703 (chr8p11.23), MYC (chr8q24.2), and CCNE1 (chr19q12). These findings suggest that ctDNA could serve as a valuable tool for early detection, molecular profiling, and disease monitoring in underserved global populations, potentially improving outcomes where traditional diagnostics are limited.

Asian populations are similarly underrepresented in breast cancer genomic research. In a large cohort of 4079 Chinese women with breast cancer, the most common somatic mutations were TP53 (49.9%), PIK3CA (30.1%), GATA3 (10.0%), NF1 (6.0%), and MAP3K1 (5.4%), with TP53 and BRCA1 mutations enriched in TNBC. Comparisons to White cohorts revealed increased NF1 and TP53 mutations in HR+/HER2− disease, and compared to patients of African ancestry, Chinese patients had higher rates of PIK3CA and lower rates of GATA3 mutations^52^.

Comparably, a retrospective study from India analysed the genomic mutational profile of 106 patients with advanced breast cancer using a ctDNA NGS assay (Guardant360). The most frequent SNVs were found in PIK3CA (40%), ESR1 (25%), and BRCA1/2 (22.6%), with amplifications in FGFR1 (13%) and EGFR (12%), and rare fusion events in genes like EML4-ALK. Remarkably, 18% of PIK3CA and 11% of ESR1 mutations occurred outside the hotspot loci^53^. In another study from India consisting of 40 patients with early-stage HR+ breast cancer, the most common mutations were PIK3CA (38%), ESR1 (34.5%), TP53 (28.1%), BRCA1 (21.8%), and BRCA2 (25%). It showed significant enrichment of circulating variants of *ESR1*, *NF1*, *ARID1A*, and *CCND1/2*, which are frequently present in patients with MBC and uncommon in those with early-stage disease. Also, a few underreported PIK3CA mutations (e.g., p.E81K, p.Q546H, p.G1049R) impacting the helical and kinase domains were detected as circulating variants in early-stage breast cancer^54^.

These population-specific genomic differences highlight the importance of regionally and ethnically diverse datasets to guide ctDNA interpretation. Unfortunately, implementation of ctDNA in LMIC faces challenges due largely to available infrastructure and resources^55^.

## Barriers to equitable implementation of ctDNA testing across racial and ethnic groups

Despite the promise of ctDNA testing in precision oncology, key barriers have limited its equitable adoption among racially and ethnically diverse populations, including cost and insurance coverage, limited access to advanced molecular diagnostics, mistrust in the healthcare system, and inadequate representation of minority groups in clinical research. Collectively, these structural and systemic issues contribute to disparities in the utilization of ctDNA and other genomic tools, ultimately limiting the reach and effectiveness of precision oncology in populations that already experience worse cancer outcomes.

Genomic tools such as NGS and assays like Oncotype DX remain cost-prohibitive for many patients, with inconsistent insurance coverage further limiting access^56,57^. Insurance disparities have been shown to affect the use of genomic testing, with White patients more likely to receive genotype-matched therapies than patients from racial and ethnic minority groups, even when mutation profiles are similar^58^. This uneven access to genomic testing directly impacts the timely adoption of ctDNA technologies in these populations.

Geographic inequities also compound these challenges. Molecular diagnostics are largely concentrated in academic medical centers, limiting accessibility for nonacademic and rural practices^59^. As a result, non-White patients—who are less likely to seek second opinions at academic institutions, according to some studies—may face additional logistical barriers to obtaining ctDNA testing. The lack of ubiquitous availability of molecular testing contributes to treatment delays and underutilization of precision oncology approaches.

Mistrust of the healthcare system remains a significant barrier, particularly among Black and other historically marginalized communities. Rooted in a legacy of unethical research practices and perpetuated by ongoing structural inequities, this mistrust can impact both clinical trial participation and the adoption of newer technologies such as ctDNA testing^24,60^. Similar patterns have been observed in germline testing for hereditary breast and ovarian cancer, where mistrust has contributed to lower uptake among Black women^61,62^. Without culturally tailored outreach and engagement strategies, these communities risk continued exclusion from advances in personalized cancer care.

Importantly, racial and ethnic minorities remain underrepresented in precision oncology trials^60,63^. This lack of diversity may limit the generalizability of assay performance and may obscure population-specific differences in ctDNA biology, shedding, and response to targeted therapy. For example, genomic databases and trial cohorts informing widely used assays, such as the 21- and 70- gene signature assays, have been composed primarily of White participants^20,64^. This raises concerns about the accuracy and applicability of these tools when used in racially diverse populations. Black, Hispanic, and Native American populations are underrepresented relative to their proportionate cancer incidence, limiting the robustness of actionable genomic data available for these groups^55,63,65^. In seminal breast cancer ctDNA studies, such as the c-TRAK TN trial, which demonstrated that post-operative ctDNA detection corresponded with high rates of metastatic disease in TNBC, race was not reported^8^.

**Fig. 1:**
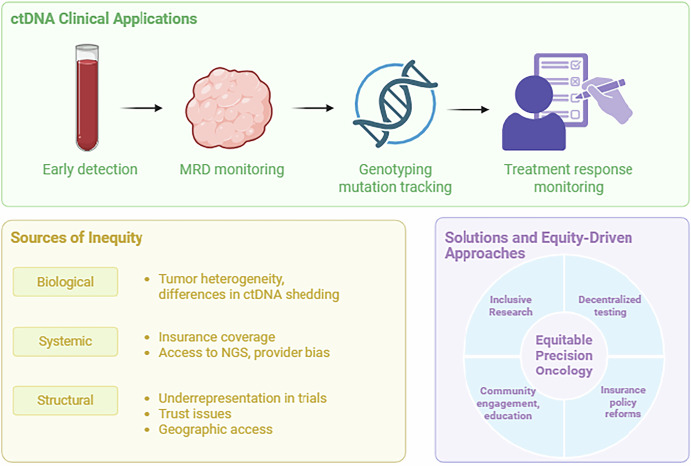
Clinical Applications, Barriers, and Equity Strategies for ctDNA-based Precision Oncology.

To close these gaps, efforts must prioritize equitable insurance coverage for genomic testing, expand the availability of ctDNA platforms in diverse care settings, and invest in trust-building through community engagement. It is critical that future ctDNA trials must intentionally include racially and ethnically diverse participants to ensure that precision oncology benefits are accessible and relevant to all patients. We summarize ongoing challenges to the equitable implementation of ctDNA in clinical practice and potential solutions in Fig. 1.

## Future Directions and Conclusions

The integration of ctDNA into cancer diagnostics and treatment personalization represents a transformative advance in precision oncology. Its potential to improve early detection, monitor MRD, and guide personalized therapies is increasingly evident. However, realizing the full benefits of ctDNA requires deliberate attention to equity in research, validation, and clinical application. Currently, racially and ethnically diverse populations remain significantly underrepresented in liquid biopsy studies, raising concerns about the generalizability of findings and equitable effectiveness of precision oncology approaches.

Addressing these disparities begins with inclusive research, and clinical utility studies of ctDNA assays must intentionally recruit diverse participants, ensuring adequate representation of population-specific variations in tumor biology, mutation patterns, and ctDNA kinetics. Without this inclusion, ctDNA-based tools risk reinforcing existing disparities rather than closing them. Broad representation will enhance the accuracy, reliability, and applicability of ctDNA-driven clinical decisions across all patient populations.

In addition to diverse enrollment, race-conscious approaches to assay development and validation should be explored. Standard ctDNA thresholds developed in predominantly White cohorts may not account for biologic differences that affect ctDNA shedding or clearance. If left unexamined, such uniform thresholds could lead to inappropriate risk stratification and missed therapeutic opportunities for minority patients. Future ctDNA research must explicitly evaluate how race, ancestry, and social determinants of health influence assay performance and clinical outcomes.

Expanding access to ctDNA testing is equally critical. Financial barriers remain a major limitation, particularly for historically marginalized populations. Strategies to expand insurance coverage, reduce testing costs, and promote equitable reimbursement policies will be essential for increasing the affordability and adoption of ctDNA-guided care. These policy changes must be paired with system-level efforts to decentralize molecular diagnostics, integrating ctDNA testing and related clinical trials into community hospitals, safety-net healthcare systems, and rural clinics.

In summary, ctDNA holds great promise for advancing personalized cancer care. However, its full impact depends on ensuring equity is integrated throughout the continuum—from research design and trial enrollment to clinical validation, reimbursement, and implementation. By addressing financial, geographic, and systemic barriers while prioritizing inclusive participation, ctDNA has the potential to evolve into not only a tool for precision but a driver of more equitable cancer care delivery.

## Data Availability

No datasets were generated or analysed during the current study.
